# F-box proteins at the crossroads of ubiquitination and tumor immunity: regulatory networks and immunotherapy strategies

**DOI:** 10.3389/fimmu.2025.1596344

**Published:** 2025-06-04

**Authors:** Mingzheng Dai, Shimin Chen, Yuanjing Wang, Jinxuan Fan, Xin Pan, Chenhui Sang, Yuchen Liu, Ming Hu, Leina Ma, Shasha Wang

**Affiliations:** ^1^ Department of Oncology, Cancer Institute, The Affiliated Hospital of Qingdao University, Qingdao, China; ^2^ Qingdao Cancer Institute, The Affiliated Hospital of Qingdao University, Qingdao University, Qingdao, China; ^3^ School of Basic Medicine, Qingdao University, Qingdao, China; ^4^ Department of Respiratory and Critical Care Medicine, Shandong Provincial Hospital Affiliated to Shandong First Medical University, Jinan, Shandong, China; ^5^ Department of Special Medicine, School of Basic Medicine, Qingdao University, Qingdao, China

**Keywords:** F-box protein, ubiquitination, cancer, immunotherapy strategies, proteasomal degradation

## Abstract

As critical substrate-recognition subunits of the SCF (SKP1-CUL1-F-box) ubiquitin ligase complex, F-box proteins mediate the ubiquitination and subsequent degradation of specific target proteins, playing pivotal roles in cell cycle regulation, signal transduction (e.g., MAPK and NF-κB pathways), and immune homeostasis. F-box proteins have dual regulatory functions in tumorigenesis and immune escape. On one hand, their expression is dynamically modulated by upstream signaling pathways (including PI3K/AKT and Wnt/β-catenin cascades) and epigenetic modifications (such as DNA methylation and histone acetylation), thereby influencing the stability of oncogenic factors (e.g., c-MYC, Cyclin E) or tumor suppressors (e.g., p53). On the other hand, F-box proteins directly regulate tumor immune microenvironments by targeting immune-related molecules for degradation, thereby modulating T-cell activation, macrophage polarization, and immune checkpoint functionality (specifically PD-1/PD-L1 axis and CTLA-4 signaling). This review systematically summarizes the upstream and downstream regulatory networks of F-box proteins, with an emphasis on their molecular mechanisms in tumor immunosuppression. It highlights the potential strategies and drug resistance mechanisms in targeting F-box proteins for combination with immunotherapies, while also discussing future research applications and development directions of F-box proteins. These insights aim to advance the development of novel immunotherapeutic strategies for precision cancer treatment.

## 1 Introduction

**Figure 1:**
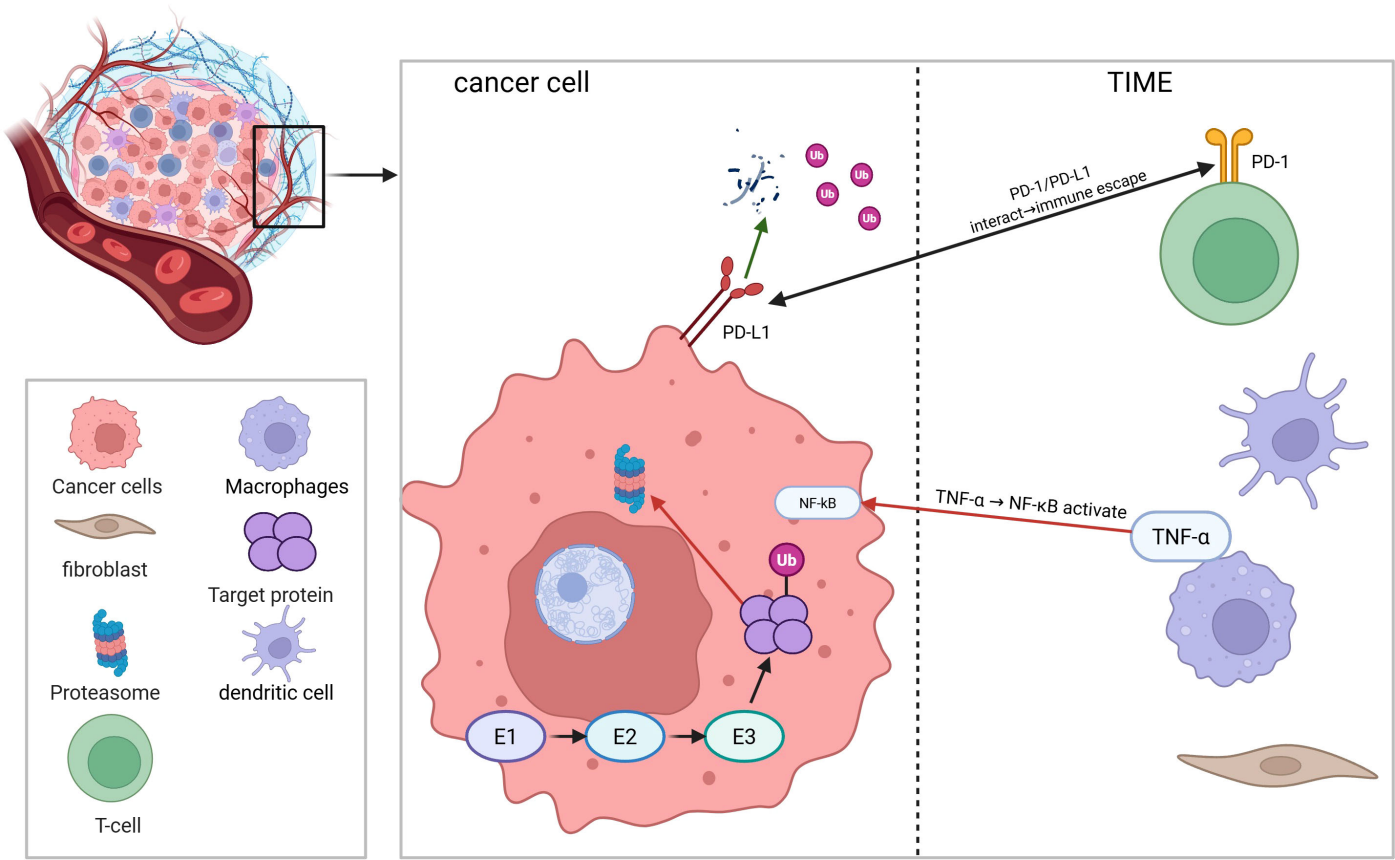
This figure was created in BioRender (https://BioRender.com). Ubiquitination regulates the crosstalk between tumors and the tumor microenvironment.

The F-box protein family represents a conserved superfamily characterized by the signature F-box domain, which is widely distributed among eukaryotes. As critical regulatory components of the ubiquitin-proteasome system (UPS), members of this family participate in essential biological processes, including cell cycle regulation, transcriptional control, apoptosis, and signal transduction, through the specific recognition and ubiquitination of substrate proteins. Recent studies have revealed the functional heterogeneity of F-box proteins in tumor development, classifying them into three major categories based on their roles: tumor-suppressive F-box proteins, proto-oncogenic F-box proteins, and context-dependent F-box proteins (1). The tumor immune microenvironment (TIME) is a key determinant of patient prognosis and treatment response. It consists of tumor cells, tumor infiltrating lymphocytes (TILs), natural killer cells (NK cells), tumor-associated non-immune cells (such as fibroblasts, fat cells) and extracellular matrix (ECM). TIME plays a central role in tumorigenesis, metastasis and immune escape. Its immune characteristics are now recognized as “cancer markers” with both prognostic prediction and treatment guidance value (2–4). Interestingly, the F-box protein family shows significant microenvironment plasticity, with their molecular behavior dynamically adapting to tumor microenvironmental factors such as hypoxia, pH fluctuations, and inflammatory cytokine stimulation. For example, under specific stress conditions, certain F-box proteins with tumor-suppressive functions may acquire pro-oncogenic properties through conformational changes or post-translational modifications. This functional switch often involves crosstalk with key signaling pathways, such as HIF-1α and NF-κB. In addition, at the molecular level, F-box proteins primarily function as substrate recognition modules within the SCF E3 ubiquitin ligase complex, mediating the ubiquitination and degradation of target proteins. Their roles in tumor regulation include cell cycle regulation, inactivation of pro-apoptotic factors and activation of oncogenic signaling pathways. This precise “molecular switch” function enables F-box protein to contribute to new immunotherapy strategies such as combined immune checkpoint inhibitors (ICIs), optimization of adoptive cell therapy, and combinations of metabolic interventions and apparent regulation (**Figure 1**).

The complexity of the F-box protein regulatory network in tumorigenesis and development has been gradually revealed, but the blind spots in key mechanisms still restrict the development of precise targeting strategies. Existing studies have not yet systematically analyzed the dynamic regulatory rules of substrate recognition among F-box family members. Although specific recognition mediated by domains such as WD40 or LRR has been recognized, more than 70% of F-box proteins still lack a complete substrate lineage map (such as the molecular interface for FBXO32 to regulate cyclinD1 has not been clarified) (5). More importantly, the regulatory dimensions of this type of E3 ligase in the tumor immune microenvironment are seriously underestimated, and the mechanisms of mediating immune escape by reshaping immune cell metabolism (such as T cell mitochondrial function) or regulating immune checkpoint molecules (such as PD-L1/CTLA-4) need to be verified. At the same time, research that breaks through the traditional K48-linked ubiquitination paradigm shows that FBXO32 can stabilize substrates through K27 ubiquitination (6), suggesting that F-box protein may constitute a new “ubiquitin code” decoding system, but its time-specific regulatory network and biological significance are still unknown. In response to these key scientific issues, future research needs to build a multi-dimensional analytical platform: to clarify the tumor type-specific regulation laws of F-box protein through single-cell multiomics technology; to develop PROTAC degradants based on structural biology to selectively intervene in pathological substrate interactions; to establish a dynamic model of immune microenvironment, and systematically analyze the new mechanisms of F-box protein to regulate anti-tumor immunity through metabolic reprogramming (such as PKM2 phosphorylation) and immune checkpoint “post-translational modification switch”. These breakthroughs will promote the transformation of F-box protein from basic research to targets for precise immunotherapy. In the future, we will also pay attention to the role of these protein families in the development of tumors, and provide more therapeutic strategies for the clinical immunotherapy of tumors.

## 2 Structure, classification, and evolution of the F-box proteins

The evolutionary pattern of the F-box gene family primarily follows the birth-death model, characterized by gene duplication and loss events. However, evidence also suggests instances of co-evolution among certain F-box genes, reflecting functional interactions or compensatory mechanisms. While some F-box genes exhibit signs of adaptive evolution in specific lineages, the majority of the family members are subject to strong purifying selection, indicative of their conserved functional roles. Population genetic analyses further reveal that domain regions within F-box genes experience significantly stronger purifying selection compared to non-domain regions, highlighting the critical functional importance of these conserved structural elements (7).

The F-box protein family exhibits remarkable diversity across eukaryotic species, with significant variations in gene number and conservation patterns. The complete genome of Saccharomyces cerevisiae encodes 11 F-box proteins, while Caenorhabditis elegans, Drosophila melanogaster, and humans possess 326, 22, and at least 38 F-box proteins, respectively. Notably, no F-box proteins have been identified in prokaryotes, underscoring their exclusive role in eukaryotic biology. Throughout eukaryotic evolution, F-box motifs have been recurrently integrated into existing proteins, contributing to functional diversification (8). More than half of the predicted C. elegans F-box protein was found along with another motif called DUF38 (unknown functional domain 38) or FTH (FOG-2 homology) (9).

The F-box domain, typically located at the N-terminus, functions in concert with C-terminal motifs to mediate protein-protein interactions. This domain, comprising approximately 40–50 amino acids, exhibits limited sequence conservation, with only a few key residues being conserved across species: leucine or methionine at position 8, proline at position 9, isoleucine or valine at position 16, leucine or methionine at position 20, and serine or cysteine at position 32 (8). Functionally, the F-box domain serves as the binding site for Skp1 or Skp1-like proteins within the SCF ubiquitin ligase complex.

According to the HUGO Gene Nomenclature Committee (HGNC), the total number of confirmed F-box protein genes is approximately 69. They are classified into three major subfamilies—FBXL, FBXW, and FBXO—based on their C-terminal secondary structures, which play a critical role in mediating specific interactions between F-box proteins and their substrate proteins.

FBXL Proteins: Characterized by leucine-rich repeats (LRRs) at their C-terminus, this subfamily comprises 22 members. FBXL proteins are involved in regulating multiple signaling pathways and exert multifaceted control over the cell cycle (10).

FBXW Proteins: Defined by the presence of WD40 repeats at their C-terminus, this subfamily includes 10 members. FBXW proteins primarily function within the SCF E3 ubiquitin ligase complex, targeting substrate proteins involved in cell cycle regulation and tumorigenesis for ubiquitination and degradation (11).

FBXO Proteins: FBXO is the largest subfamily of F-box proteins, with 38 members currently known. The “O” in FBXO denotes “other,” referring to proteins with diverse C-terminal secondary structures that do not fall into the FBXL or FBXW categories. As core components of E3 ubiquitin ligase complexes, FBXO proteins play pivotal regulatory roles in a wide range of cellular processes (12).

## 3 F-box proteins that affect immunity

### 3.1 β-TrCP

The core function of β-TrCP is to regulate NF-κB and Wnt signaling pathways. On the one hand, β-TrCP activates NF-κB signaling by degrading IκB (NF-κB inhibitors), promotes the release of pro-inflammatory factors (such as TNF-α, IL-6), and enhances the survival and metastasis of tumor cells (13, 14). On the other hand, β-TrCP mediates the ubiquitination and degradation of β-catenin to inhibit the classical Wnt pathway, but its dysfunction leads to the accumulation of β-catenin, promoting epithelial-mesenchymal transition (EMT) and the formation of an immunosuppressive microenvironment. For example, high expression of β-catenin is associated with reduced CD8+T cell infiltration, suggesting that the β-TrCP-Wnt axis may affect the response to immune checkpoint treatment by regulating immune cell infiltration. In addition, pan-cancer analysis has shown that the expression of FBXW1 (β-TrCP) was significantly negatively correlated with immune score and matrix score, and positively correlated with tumor purity. In lung cancer (LUAD) and renal cancer (KIRC), high expression of FBXW1 is associated with reduced infiltration of immunocompetent cells such as NK cells and CD8+T cells, suggesting that it may promote immunosuppression in the microenvironment by inhibiting anti-tumor immune responses (14).

Metabolic abnormalities are important drivers of tumor immune escape. Wei Wenyi’s team found that β-TrCP degrades Lipin1, a key enzyme in lipid metabolism, through ubiquitination, regulating liver lipid metabolism homeostasis. Lipid overload can induce polarization of M2-type macrophages and inhibit T cell function, while β-TrCP-mediated disorders of lipid metabolism may affect antigen presentation efficiency by altering cell membrane components, thereby weakening adaptive immune responses (13, 14). In pancreatic cancer, the low-sugar and hypoxia microenvironment promotes tumor progression by activating HIF1 α and c-MYC signals, and β-TrCP may indirectly regulate these pathways. For example, BZW1, a gene related to the function of β-TrCP, enhances glycolytic metabolism steadily by stabilizing HIF1 α and c-MYC, exacerbating the formation of an immunosuppressive microenvironment. In addition, the interaction between β-TrCP and Akt is particularly significant under hypoxic conditions: hypoxia induces Akt hydroxylation and inhibits its activity, while β-TrCP may affect the survival and angiogenesis of tumor cells by regulating Akt stability, further promoting the secretion of immunosuppressive angiogenesis factors (such as VEGF) (15).

The β-TrCP dysfunction is closely related to tumor immune escape and treatment resistance. In HER2-positive breast cancer, the new β-TrCP-343aa isomer encoded by circular RNA (circ-β-TrCP) binds competitively to NRF2, blocking its ubiquitination and degradation, resulting in over-activation of NRF2. High expression of NRF2 not only enhances the antioxidant capacity of tumor cells, but also promotes immune escape by inhibiting T cell function, ultimately leading to trastuzumab resistance (15). In addition, β-TrCP affects immune checkpoint function by regulating PD-L1 expression. Studies have shown that the expression of β-TrCP is negatively correlated with PD-L1, while FBXW7 (another F-box protein) is positively correlated with the infiltration of immunocompetent cells, suggesting that members of the β-TrCP family have a heterogeneous role in the regulation of immune checkpoints.

The expression level of β-TrCP is significantly correlated with clinical outcomes in multiple tumors. In breast cancer, high expression of β-TrCP is associated with estrogen receptor positivity, lymph node metastasis and poor prognosis, and the 5-year survival rate of patients with positive results is significantly reduced. In gliomas, low expression of β-TrCP and high expression of Bmi-1 jointly drive malignant progression, suggesting their potential as a joint prognostic marker. Strategies for targeting β-TrCP include inhibiting its substrate degradation function or using its degradation mechanism to clear immunosuppressive molecules. For example, inhibition of eIF2 α phosphorylation down-regulates BZW1 expression and significantly inhibits pancreatic cancer cell growth (16). In CAR-T therapy, regulating β-TrCP may improve efficacy by enhancing T cell activity or degrading PD-L1. The combination of β-TrCP inhibitors and immune checkpoint blockers such as anti-PD-1 antibodies has potential synergistic effects. For example, inhibiting β-TrCP-mediated stabilization of NRF2 enhances oxidative stress-induced tumor cell death while reversing the immunosuppressive microenvironment.

### 3.2 FBXW7

In cancer, FBXW7 plays a critical role in modulating the tumor immune microenvironment by inhibiting macrophage M2 polarization through the targeted degradation of c-MYC. M2 macrophages, a pro-tumorigenic phenotype, contribute to cancer progression by promoting angiogenesis, enhancing tumor cell invasiveness, remodeling the extracellular matrix, and facilitating tumor cell proliferation. By mediating c-MYC degradation, FBXW7 disrupts the signaling pathways that drive M2 polarization, thereby suppressing the tumor-promoting functions of these macrophages. This regulatory mechanism highlights the potential of FBXW7 as a key modulator of cancer progression through its influence on the tumor immune microenvironment (17).

FBXW7 further regulates the tumor immune microenvironment by targeting C/EBPδ for degradation. This process suppresses the expression of Toll-like receptor 4 (TLR4), leading to a reduction in inflammatory responses and a modulation of the innate immune response of macrophages to pathogenic stimuli. By controlling C/EBPδ stability, FBXW7 acts as a critical regulator of macrophage-mediated immunity, highlighting its dual role in both tumor suppression and immune modulation (18).

The tumor microenvironment can also be altered through the process of epithelial-mesenchymal transition (EMT), in which epithelial cells transform into migratory and invasive cells (19). EMT causes individual cancer cells to spread from the primary tumor site, participate in the dedifferentiation process that leads to malignancy, and increase the malignancy of the tumor (20). Some proteins regulated by FBXW7 affect EMT, such as EMT transcription factors (EMT-TFs), including mTOR, Snai1, c-MYC (21, 22), SOX9 (23, 24), and ZEB2 (25). Studies have also shown that activation of CCL2 (C-CMotifChemokineLigand2) can promote the formation of the metastatic niche by recruiting monocyte myeloid-derived suppressor cells and macrophages (26, 27).

FBXW7 can inhibit the transcriptional activation of the CCL2 gene by negatively regulating the expression level of NOTCH; inhibition of CCL2/CCR2 signal transduction has been shown to inhibit tumor metastasis, indicating that the FBXW7/NOTCH/CCL2 axis plays an important role in regulating cancer metastasis (28). In addition, studies have also found that FBXW7 can control the expression of CCL2 by stimulating the ubiquitination of EZH2 (enhancer of zeste homolog 2), thereby reducing global H3K27me3 on the CCL2 promoter (29).

FBXW7 plays a critical role in regulating innate immune signaling pathways. Loss of FBXW7 impairs the cellular ability to activate double-stranded RNA (dsRNA) and interferon (IFN) responses, which are crucial for antiviral defense and often triggered in cells harboring oncogenic stimuli. This defect is associated with the downregulation of key cytosolic RNA sensors, including MDA5 (melanoma differentiation-associated protein 5) and RIG-I (retinoic acid-inducible gene I). The diminished dsRNA sensing capacity in FBXW7-deficient cells has been linked to resistance to anti-PD-1 immunotherapy, underscoring the importance of FBXW7 in maintaining immune surveillance and response to cancer therapies (30).

Loss of FBXW7-mediated proteasomal degradation of interferon-γ receptor 1 (IFNGR1) results in sustained activation of IFNGR1 signaling. This aberrant signaling stimulates the recruitment of immunosuppressive neutrophils to tumor sites, fostering tumor growth and metastasis in patients with triple-negative breast cancer (TNBC) (31).

FBXW7 plays a critical role in inducing the degradation of nuclear factor of activated T cells 1 (NFAT1) in renal cancer cells, a process regulated by glycogen synthase kinase-3β (GSK-3β). Overexpression of FBXW7 reduces NFAT1 protein levels, while inhibition of GSK-3β increases NFAT1 stability. The consensus motif (T/S)PXX(S/T/D/E) recognized by FBXW7 overlaps with the GSK-3β phosphorylation site, which is located downstream of the PI3K/AKT signaling axis. This regulatory mechanism underscores the importance of FBXW7 in controlling NFAT1 levels and its potential role in renal cancer progression (32).

**Figure 2:**
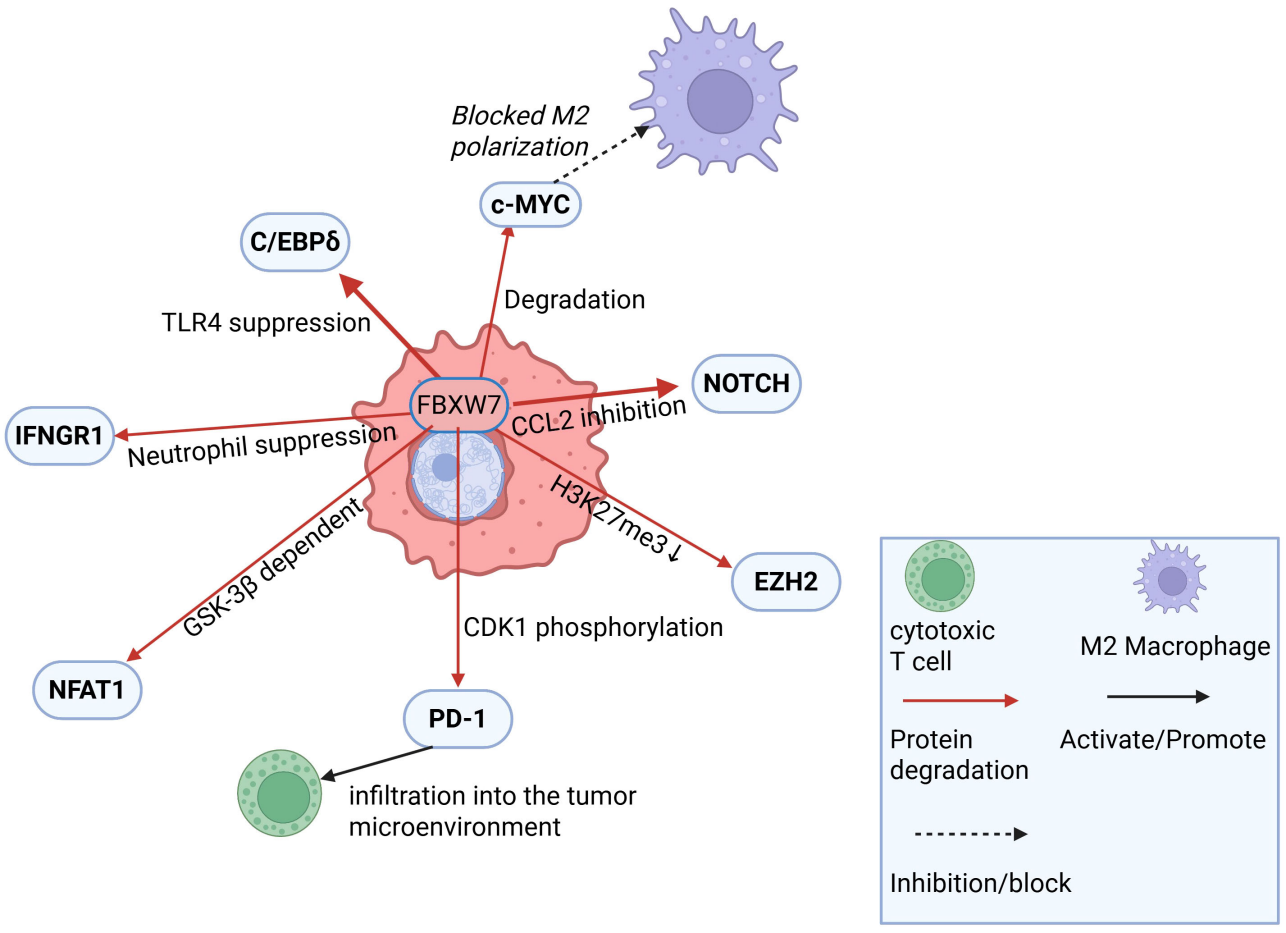
This figure was created in BioRender (https://BioRender.com). FBXW7 regulates tumor immunity via Ubiquitin-Dependent signaling.

FBXW7 functions as an E3 ubiquitin ligase for programmed cell death protein 1 (PD-1), promoting its K48-linked polyubiquitination at Lys233 in a phosphorylation-dependent manner. This ubiquitination leads to PD-1 degradation, enhancing cytotoxic lymphocyte infiltration into the tumor microenvironment and impairing the sensitivity of non-small cell lung cancer (NSCLC) to anti-PD-1 immunotherapy. Cyclin-dependent kinase 1 (CDK1)-mediated phosphorylation of the Ser261 residue facilitates PD-1 nuclear translocation and its subsequent binding to FBXW7. High FBXW7 expression is associated with a “hot” tumor microenvironment, which exhibits a more favorable response to PD-1 blockade therapy, highlighting FBXW7 as a potential biomarker for immunotherapy efficacy (33) (**Figure 2**).

#### 3.2.1 FBXW7 upstream factors

A network of upstream factors tightly regulates FBXW7, including p53, C/EBP-δ, EBP2, Pin1, Hes-5, and Numb4. These regulators modulate FBXW7 expression and activity, influencing its role in key cellular processes such as cell cycle control, apoptosis, and tumor suppression.

##### 3.2.1.1 p53

The p53 tumor suppressor protein is a well-established master regulator in the majority of human cancers, playing critical roles in cell growth, DNA synthesis and repair, differentiation, apoptosis, and cellular stress responses. These stress responses include heat shock, hypoxia, osmotic shock, and DNA damage, underscoring p53’s central role in maintaining genomic stability and preventing tumorigenesis (34).

Recent studies have identified FBXW7 as a direct transcriptional target of p53 (35), further expanding the p53 tumor suppressor network. Kimura et al. demonstrated that adenovirus-mediated transfer of wild-type p53 into p53-deficient cells dramatically upregulates FBXW7 expression. They identified a functional p53-binding site within the first exon of FBXW7, confirming its p53-dependent transcriptional activity (36).

Building on this discovery, Mao et al. revealed that FBXW7 mediates p53’s critical role in the DNA damage response, providing compelling evidence that FBXW7 functions as a p53-dependent tumor suppressor gene in oncogenesis (37). These findings highlight the intricate interplay between p53 and FBXW7 in tumor suppression and stress response pathways.

##### 3.2.1.2 C/EBP-δ

C/EBP-δ, one of six isoforms in the C/EBP (CCAAT/enhancer-binding protein) family, plays a critical role in regulating the growth and differentiation of various cell types (38). In cancer biology, reduced expression of C/EBP-δ due to promoter methylation has been observed in breast cancer cell lines and primary breast tumors, suggesting its potential role as a tumor suppressor (39).

Recent studies have revealed a novel regulatory mechanism involving C/EBP-δ and FBXW7. C/EBP-δ directly inhibits FBXW7 expression, leading to enhanced mTOR/Akt/S6K1 signaling and increased translation and activity of hypoxia-inducible factor 1α (HIF-1α). This regulatory axis is particularly significant, as both mTOR and HIF-1α are established substrates of FBXW7, highlighting the critical role of C/EBP-δ in modulating oncogenic signaling pathways through FBXW7 suppression (40).

##### 3.2.1.3 EBP2

EBP2 is a small nucleolar protein essential for mammalian cell proliferation (41). In 2011, Welcker et al. discovered that EBP2 directly interacts with FBXW7, regulating its nucleolar localization. This interaction suggests a novel mechanism by which EBP2 may influence FBXW7 function within the nucleolus. Further studies by Clurman et al. revealed that EBP2 acts as a pseudosubstrate, targeting FBXW7 to the nucleolar compartment. This finding provides insight into the spatial regulation of FBXW7 activity, although the precise role of EBP2 in tumorigenesis remains to be fully elucidated (42).

##### 3.2.1.4 Pin1

Pin1 is a unique peptidyl-prolyl isomerase that specifically catalyzes the isomerization of phosphorylated Ser/Thr-Pro peptide bonds, inducing conformational changes in target proteins with high efficiency. Dysregulation of Pin1 has been implicated in various diseases, including Alzheimer’s disease, aging-related disorders, and cancer (43).

Recent studies have revealed that Pin1 acts as an upstream regulator of FBXW7, influencing the stability of several FBXW7 substrates, such as Mcl-1 and c-Jun (44). Experimental evidence demonstrates that Pin1 interacts with FBXW7 in a phosphorylation-dependent manner and negatively regulates its stability by promoting FBXW7 self-ubiquitination and subsequent degradation. This Pin1-mediated reduction in FBXW7 expression contributes to tumorigenesis, highlighting Pin1 as a potential therapeutic target in cancer (45).

##### 3.2.1.5 Hes-5

Hes-5, a downstream target gene of the Notch signaling pathway, plays a critical role in regulating key cellular processes such as proliferation, apoptosis, migration, invasion, and angiogenesis. The Notch pathway, a ligand-receptor signaling cascade, has been extensively implicated in tumor development and progression across various human malignancies (46–48).

Sancho et al. demonstrated that Hes-5 directly represses the transcription of FBXW7β, a key isoform of FBXW7. Furthermore, their work revealed the existence of a NICD/Hes-5/FBW7β positive feedback loop, which contributes to FBXW7 haploinsufficiency and underscores the intricate regulatory interplay between the Notch pathway and FBXW7 in cancer biology (49).

##### 3.2.1.6 Numb4

Numb has recently been recognized as a bona fide tumor suppressor in human cancers, where it modulates multiple signaling pathways, including p53, Notch, and Hedgehog (50). Among its isoforms, Numb4 plays a particularly significant role by promoting the assembly and activation of the FBXW7 ubiquitin ligase complex. This activity enhances the degradation of Notch, further underscoring Numb4’s role as a critical regulator of tumor-suppressive pathways (51).

### 3.3 SKP2

Tumor metabolic reprogramming, such as the Warburg effect, is closely related to immune escape. Liu Jiankang’s team found that SKP2 drives cell cycle-dependent metabolic oscillations by regulating the stability of IDH1/2: the TCA cycle is dominant in the G1 phase, while the S phase prefers glycolysis (52). In prostate cancer, high expression of SKP2 leads to IDH1 degradation. As a core member of the SCF ubiquitin ligase complex, SKP2 regulates cell cycle processes by ubiquitating and degrading substrates. In recent years, studies have found that inhibition of SKP2 can induce DNA replication pressure and genomic instability, thereby activating the inherent immune signaling pathway of tumor cells. Li Xiong’s team confirmed in triple-negative breast cancer (TNBC) that SKP2 inhibitors lead to DNA double strand breaks and the formation of micronucleus structures by upregulating the DNA replication permitting factors CDC6 and CDT1, activating the cGAS/STING pathway, and promoting the secretion of type I interferons (such as IFN-β) and chemokines (such as CXCL10). This process significantly increases the infiltration of CD8 +T cells in the tumor microenvironment and enhances the efficacy of PD-1 antibodies (53). It is worth noting that the tumor inhibitory effect of SKP2 deletion on immunodeficient mice is weaker than that of immunocompetent mice, indicating that its antitumor effect is highly dependent on the reshaping of the immune microenvironment. This study provides a new target for TNBC immune combination therapy, suggesting the potential value of SKP2 inhibitors combined with immune checkpoint blockers.

The function of SKP2 is regulated by a variety of post-translational modifications, among which O-GlcNAc modifications and ubiquitination modifications play an important role in tumor immune escape. Wang Kai’s team has found in liver cancer that O-GlcNAc modification at the Ser34 site of SKP2 can enhance its binding ability to SKP1, stabilize the function of the SCF complex, promote the ubiquitination and degradation of p27 and p21, accelerate G1/S phase transition and drive tumor proliferation (54). This modification also increases the half-life of SKP2 by inhibiting APC-CDH1-mediated degradation, resulting in continued uncontrolled cell cycle. In addition, Wei Wenyi’s team found that the K68/K71 acetylation modification of SKP2 can promote its cytoplasmic localization and enhance tumor metastasis by degrading E-cadherin, while SIRT3 deacetylase can reverse this process (52). It is worth noting that the modified state of SKP2 may affect the recognition of tumors by immune cells: cell cycle abnormalities may reduce antigen presentation time, while metastasis inhibition may reduce the recruitment of immunosuppressive cells such as Treg.

SKP2 directly participates in the formation of chemotherapy resistance and immunosuppressive microenvironments by regulating programmed necrosis and apoptosis-related proteins. Research from Central South University has shown that in cisplatin-resistant NSCLC cells, SKP2 degrades the necrotic apoptosis key protein MLKL through ubiquitination, inhibiting cisplatin-induced immunogenic cell death (ICD). Deletion of MLKL leads to reduced release of damage-associated molecular patterns (DAMPs), impairing dendritic cell activation and T cell infiltration (55).

Tumor metabolic reprogramming, such as the Warburg effect, is closely related to immune escape. Liu Jiankang’s team found that SKP2 drives cell cycle-dependent metabolic oscillations by regulating the stability of IDH1/2: the TCA cycle is dominant in the G1 phase, while the S phase prefers glycolysis (3). In prostate cancer, high expression of SKP2 leads to IDH1 degradation, enhances glycolysis and promotes tumor proliferation. It is worth noting that lactic acid produced by glycolysis inhibits T cell function and promotes polarization of M2-type macrophages, while inhibition of SKP2 reverses the metabolic phenotype and improves the immunosuppressive microenvironment by restoring IDH1 levels. In addition, SKP2-mediated cell cycle acceleration may reduce immunogenicity by shortening antigen processing time, while metabolic intervention may synergistically enhance immunotherapy effects.

### 3.4 FBXL2

FBXL2 is an anti-inflammatory molecule that mediates the degradation of TNF receptor-associated factor (TRAF), thereby reducing the release of inflammatory cytokines. FBXL2 recognizes Trp-73 within NALP3 for interaction and targets Lys-689 within NALP3 for ubiquitination and degradation. Unique small molecule inhibitors of FBXL2 restore FBXL2 levels, resulting in a reduction of NALP3 protein levels in cells, thereby reducing the release of IL-1 and IL-18 in human inflammatory cells following NALP3 activation. Ubiquitination of NALP3 is increased following FBXL2 expression. FBXL2 selectively binds NALP3 as it does not bind to the NALP6 inflammasome. FBXL2 efficiently catalyzes NALP3 ubiquitination. NALP3 is a molecular target for FBXL2-mediated degradation, and endotoxins inhibit this process (56).

FBXL2 was identified as a protein that regulates adipogenic and mitogenic programs. FBXL2 acts as a key pan-reactive inhibitor of TRAF function by mediating their ubiquitination and degradation in epithelial cells and human monocytes. At the same time, FBXL2 can act as an inhibitor of TRAF protein activation by mediating their cellular localization to regulate downstream kinase signaling. FBXL2 targets six TRAF family proteins (TRAF1-6) for ubiquitination and degradation, using conserved molecular features, leading to reduced cytokine secretion in inflammatory cells (57).

### 3.5 FBXO3

FBXO3 is a proinflammatory molecule that is activated by endotoxin in leukocytes. As a proinflammatory E3 ligase subunit, it facilitates the ubiquitination and subsequent degradation of the anti-inflammatory E3 ligase sub-unit FBXL2. This process is crucial in the Pseudomonas pneumonia model, where FBXO3 triggers proinflammatory effects.

Specifically, FBXO3 inhibits FBXL2’s activity by targeting it for ubiquitination and degradation, which in turn stimulates the release of cytokines. The role of endogenous FBXO3 is pivotal in mediating cytokine-driven inflammation during microbial infections. Additionally, the depletion of endogenous FBXO3 has been observed to reduce the release of certain proinflammatory mediators induced by stimuli other than endotoxin, such as Pam3CSK4 and TNF, highlighting its broader role in inflammatory responses (57).

AIRE (autoimmune regulator) interacts with the FBXO3 E3 ubiquitin ligase, forming a functional relationship within the SCFFBXO3 complex. This complex ubiquitinates AIRE, enhancing its binding to the positive transcription elongation factor b (P-TEFb) and subsequently boosting its transcriptional activity. FBXO3 plays a critical role in regulating AIRE by promoting its ubiquitination, which destabilizes AIRE while simultaneously being essential for its transcriptional activity.

The interaction between AIRE and FBXO3 is mediated by the N-terminal 207 residues of AIRE, which bind to FBXO3 in both transformed cells and host-associated tissues. Notably, AIRE and FBXO3 not only associate with each other and the broader E3 ligase complex but also colocalize in regions of active transcription, underscoring their functional synergy in transcriptional regulation.

FBXO3 interacts with IRF3 and IRF7, specifically catalyzing K27-linked ubiquitination of these transcription factors. This modification triggers their proteasomal degradation, ultimately suppressing the activity of IRF3 and IRF7. Additionally, FBXO3 promotes the ubiquitination of phosphorylated autoimmune regulators, resulting in their rapid degradation. This process facilitates the recruitment of CycT1 and P-TEFb to autoimmune regulator-responsive promoters, enhancing their transcriptional activity on thymic tissue-specific antigens. Together, these mechanisms highlight the dual role of FBXO3 in modulating immune responses through the regulation of key transcriptional regulators (58).

A recent study revealed an association between FBXO3 and SMURF1, a member of the Nedd4 family. FBXO3 mediates the degradation of SMURF1, which stabilizes key intermediates in the BMP signaling cascade. This stabilization enhances the responsiveness of downstream target genes, highlighting a novel regulatory role of FBXO3 in modulating BMP signaling pathways (59).

FBXO3 plays an inhibitory role in IFN activation induced by poly(I:C) or SVCV. It is a key regulator in cellular antiviral responses, as it suppresses the expression of critical antiviral genes and facilitates SVCV replication. Additionally, FBXO3 negatively modulates antiviral responses, functioning in a manner analogous to FBXO6 by independently regulating IFN-I signaling outside the SCF complex. Studies utilizing zebrafish cell lines and zebrafish models have elucidated the role of FBXO3 in antiviral innate immunity, offering valuable insights into its potential functions and underlying mechanisms in this context (60).

#### 3.5.1 The interaction between FBXL2 and FBXO3

There is a molecular interaction between the two F-box proteins, FBXO3 and FBXL2, which regulate cytokine secretion by modulating TRAF protein stability. FBXL2 itself is a substrate for intracellular processing by FBXO3, leading to the upregulation of TRAF protein levels and the subsequent triggering of cytokine-mediated inflammation (56). FBXO3 plays a role in reducing the immune regulatory activity of FBXL2. In MLE cells, deficiency of FBXO3 increases the half-life of FBXL2, whereas overexpression of FBXO3 destabilizes the FBXL2 protein. FBXO3 specifically targets FBXL2 by recognizing the T404 residue as a molecular tag, recruiting the SCF complex to ubiquitinate FBXL2 at the K201 site. This mechanism highlights the critical role of FBXO3 in regulating FBXL2 stability and its downstream inflammatory effects (57).

### 3.6 FBXO7

FBXO7 plays a critical role in regulating the cell cycle and metabolic processes in T cells. It exerts opposing effects on CDK6 and p27, promoting the formation of the cyclin D-CDK6 complex while stabilizing the cyclin-dependent kinase inhibitor p27. This dual regulation facilitates the proliferation of immature thymocytes. Loss of FBXO7 expression significantly reduces the differentiation of thymocytes to the single-positive (SP) stage. p27, a key mediator of T cell quiescence, is downregulated at both transcriptional and translational levels upon T cell receptor (TCR) crosslinking, leading to increased activity of cyclin E/CDK2 and cyclin A/CDK2 complexes and subsequent S-phase entry. The increased proliferation and apoptosis observed in FBXO7-deficient T cells (Fbxo7LacZ/LacZ) can be attributed to reduced p27 expression and elevated overall CDK activity (61).

FBXO7 also regulates glycolysis through its interaction with phosphofructokinase-platelet (PFKP), a key substrate of CDK6 in some T-cell acute lymphoblastic leukemia (T-ALL) cells. FBXO7 promotes CDK6 activity, which in turn inhibits PFKP and suppresses glycolysis in T cells. Notably, the interaction between FBXO7 and PFKP is independent of CDK6, whereas CDK6’s interaction with PFKP requires FBXO7. FBXO7 facilitates two post-translational modifications of PFKP: CDK6-dependent ubiquitination and phosphorylation. Cells deficient in FBXO7 exhibit reduced CDK6 activity, and hematopoietic and lymphocyte cells show significant dependence on FBXO7 expression. Despite low viability and activation, FBXO7-deficient CD4+ T cells display increased glycolysis. FBXO7 expression is glucose-responsive at both transcriptional and post-translational levels and is associated with blood glucose regulation. While FBXO7 does not affect PFKP steady-state levels, its knockdown reduces the inactive monomeric/dimeric forms of PFKP. Malignant T cells with reduced FBXO7 exhibit elevated glycolysis, indicating that FBXO7 negatively regulates glycolytic activity (62).

### 3.7 FBXO11

FBXO11 promotes the degradation of BCL6 in B-cell lymphoma through ubiquitination, playing a critical role in regulating BCL6 protein levels. The posttranscriptional regulation of FBXO11 and BCL6 expression, mediated by miR-155, may contribute to an impaired germinal center (GC) response. Furthermore, unbiased proteomic analysis of CIITA-binding proteins identified FBXO11 as a bona fide E3 ligase for CIITA. FBXO11 regulates CIITA protein levels via ubiquitination-mediated degradation, highlighting its broader role in modulating immune-related transcriptional regulators (63).

Studies have revealed that the CtBP complex transcriptionally represses MHC-II pathway genes, while FBXO11, a component of the E3 ubiquitin ligase complex, mediates the degradation of CIITA, the master transcription factor regulating MHC-II expression. These findings provide a theoretical foundation for therapeutic strategies aimed at restoring tumor-specific MHC-II expression to address AML relapse following allogeneic transplantation. Additionally, this mechanism may enhance the efficacy of immunotherapy in non-myeloid malignancies, offering a promising avenue for broader cancer treatment applications (64).

### 3.8 FBXO32

FBXO32 can indirectly or directly regulate the expression of immune checkpoint molecules such as PD-L1 through multiple signaling pathways, thereby promoting tumor immune escape. For example, in lung adenocarcinoma (LUAD), FBXO32 degrades the cancer suppressor gene PTEN through ubiquitination, removing its inhibition on the PI3K/AKT/mTOR pathway, resulting in abnormal activation of AKT/mTOR signaling. This pathway can upregulate the expression of PD-L1, inhibit the killing function of CD8 +T cells, and promote the infiltration of regulatory T cells (Tregs), forming an immunosuppressive microenvironment (65). Similarly, in esophageal cancer, DNMT1-mediated apparent silencing of FBXO32 leads to stabilization of CDK9 protein, which further suppresses T cell responses by enhancing the transcriptional activity of immune-related genes such as PD-L1nbsp;(66). These studies suggest that FBXO32 regulates immune checkpoints through epigenetic or post-translational modifications, providing a theoretical basis for combining immune checkpoint inhibitors such as anti-PD-1/PD-L1.

Tumor metabolic reprogramming is an important mechanism for the formation of immunosuppressive microenvironments. c-Myc is a core transcription factor in metabolic regulation, and its activity is strictly regulated by FBXO32. Studies have found that FBXO32 degrades c-Myc through ubiquitination, inhibiting its mediated glycolysis and glutamine metabolism, thereby reducing lactic acid accumulation in the tumor microenvironment and improving the functional failure of CD8 +T cells (67). However, there is a negative feedback loop between c-Myc and FBXO32: c-Myc can transcribically activate FBXO32, and the degradation of FBXO32 limits the pro-metabolic effect of c-Myc. This dynamic balance may explain the functional heterogeneity of FBXO32 among different tumor types. For example, in liver cancer, the high expression of FBXO32 may promote tumor proliferation by stabilizing the D-type cyclin (CyclinD), and at the same time improve metabolic inhibition by inhibiting c-Myc, suggesting that its dual effects need to be combined with specific microenvironment analysis (6).

FBXO32 can indirectly recruit immunosuppressive cells by activating specific signaling pathways. For example, in liver and pancreatic cancer, FBXO32 activates the CDK4/6-Rb pathway by stabilizing CyclinD1, promotes tumor cells to secrete the chemokine CXCL12, inhibits T cell infiltration into tumor sites, and simultaneously recruits M2-type macrophages and myeloid-derived suppressor cells (MDSCs) to form an immune “cold” tumor phenotype (6). In addition, FBXO32-mediated epithelial-mesenchymal transformation (EMT) enhances the expression of Snail and ZEB1 through CtBP1 ubiquitination in breast cancer, which not only promotes tumor metastasis, but also upregulates PD-L1 and attracts MDSCs, further weakening the anti-tumor immune response (65). These mechanisms suggest that targeting FBXO32 may enhance immunotherapy efficacy by reshaping immune cell infiltration patterns.

### 3.9 FBXO38

FBXO38 ubiquitinates fibrinogen-like protein 1 (FGL1), negatively regulating its stability and thereby inhibiting tumor immune evasion. Loss of FBXO38 significantly increases FGL1 abundance, which suppresses CD8+ T cell infiltration, enhances tumor growth, and promotes immune escape. Conversely, FBXO38 restoration enhances tumor sensitivity to anti-FGL1 therapy and amplifies the inflammatory response. Clinically, FBXO38 levels are negatively correlated with FGL1 and interleukin-6 (IL-6), while FGL1 and IL-6 levels are positively associated with advanced TNM stages. In contrast, FBXO38 and CD8+ T cell infiltration are negatively correlated with TNM stage, highlighting FBXO38’s role in modulating tumor progression and immune responses (68).

Furthermore, FBXO38 deficiency leads to increased lysosomal degradation of STING protein, inhibiting the activation of the cGAS-STING pathway. This results in reduced secretion of downstream effectors such as IFNA1 and CCL5, ultimately promoting tumor proliferation. FBXO38 also modulates CD8+ T cell infiltration *in vivo* through the interferon-α response pathway, further underscoring its importance in anti-tumor immunity (69). To sum up, the above Targets and biological functions of the F-box protein family are shown in (**Table 1**)

**Table 1:** Targets/substrates of F-box proteins and their biological functions.

F-box proteins	Targets/substrates	Biological functions
β-TrCP(FBXW1)	β-catenin, I κB	Activate the NF-κB pathway by degrading I κB, proinflammatory tumor microenvironment; regulate Wnt/β-catenin signaling to promote tumor cell invasion
FBXW7	NOTCH, c-MYC, C/EBPδ, IFNGR1	Inhibit the transcriptional activation of CCL2 gene; Inhibit the of polarization macrophage M2 polarization; Modulate the tumor immune microenvironment; Recruit immunosuppressive neutrophils to the tumor site
SKP2(FBXL1)	p27, p21	Promote the accumulation of cyclin (such as CyclinD/E) and drive tumor cell proliferation; targeted degradation of p27 accelerates G1/S phase conversion
FBXL2	Trp-73, TRAF	Perform ubiquitin linking and degradation; Regulate kinase signaling
FBXO3	AIRE, SMURF1	Increased its binding to positive transcription extension factor b(P-TEFb) and enhance its transcriptional activity; Stabilize intermediates in the BMP signaling cascade, thus improve the responsiveness of target genes
FBXO7	CDK6, p27, PFKP	Play a role in cell cycle regulation; Affect glycolysis in T cells
FBXO11	BAHD1, SNAIL, Cdc25A	Degradation of BAHD1 relieves inhibition of erythroid genes by PRC2 complex; targeting SNAIL inhibits EMT and metastasis; degradation of Cdc25A inhibits proliferation and glycolysis of glioblastoma
FBXO32	Cyclin D1/D2/D3	Ubiquitination stabilizes D-type cyclin through K27, activates the CDK4/6- Rb pathway and drives tumor growth; interferes with its axis can enhance the efficacy of CDK4/6 inhibitors
FBXO38	FGL1	suppresses CD8+ T cell infiltration, enhances tumor growth, and promotes immune escape

## 4 MiRNA and F-box

**Figure 3:**
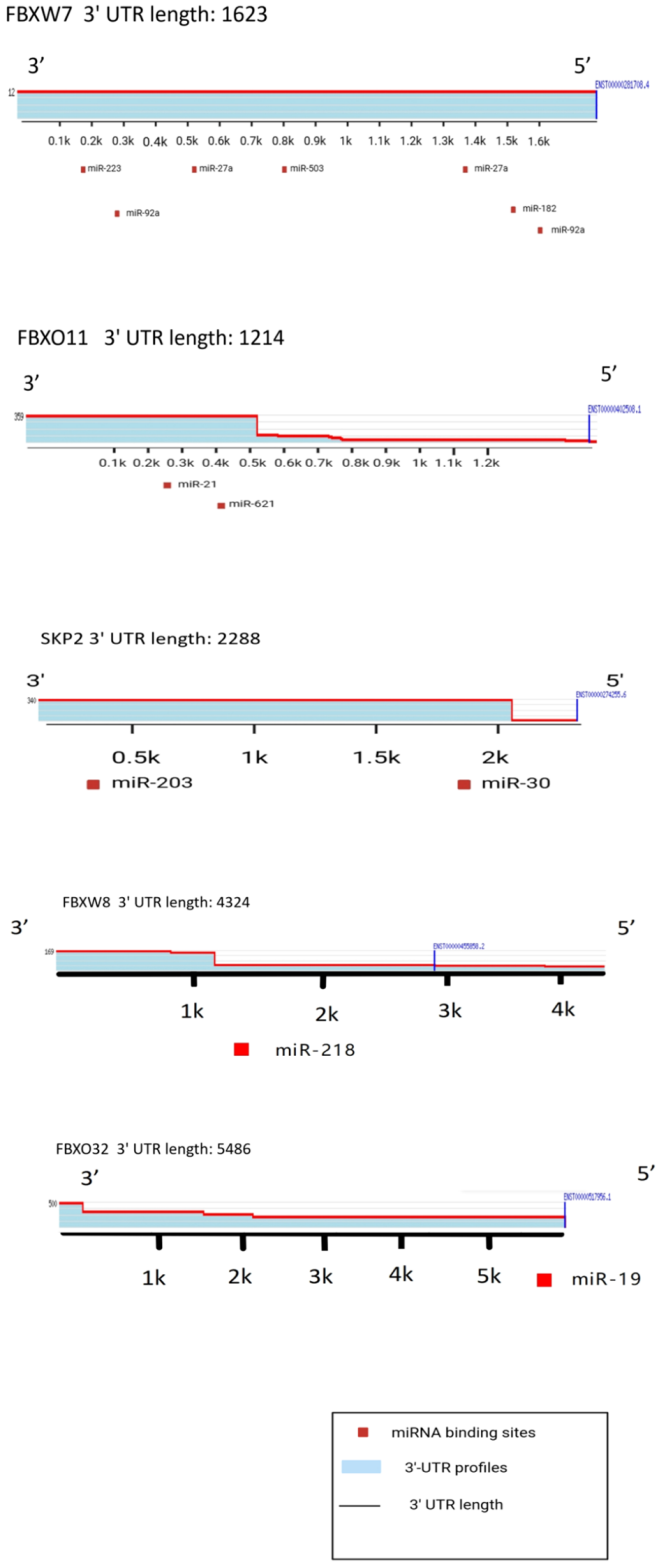
Related miRNA that affect F-box protein expression and their binding sites on 3’-UTR.

Each miRNA can fine-tune the expression of multiple target genes, so multiple F-box proteins may be inhibited by the same miRNA. At the same time, one F-box protein may be regulated by multiple miRNAs in different types of cancer (**Figure 3**).

### 4.1 FBXW7

MiR-27a negatively regulates FBXW7 expression in mouse primary erythrocytes (70). This miR-27a-dependent repression of FBXW7 may facilitate the G1-S phase transition by inhibiting FBXW7 and alleviating its suppression of cyclin E (42). FBXW7 has been identified as a direct target of miR-27a in colon cancer cells (71), where upregulation of miR-27a promotes cell proliferation, likely due to the inhibition of FBXW7 expression and subsequent elevation of cyclin E levels. Similar observations have been reported in human lung cancer specimens and childhood B-cell acute lymphoblastic leukemia cells. Additionally, miR-182 and miR-503 are sequentially upregulated in colon cancer cells and synergistically target FBXW7 for genetic repression (72), further highlighting the role of microRNAs in modulating FBXW7 expression and its downstream effects on cell cycle regulation and cancer progression.

### 4.2 FBXO11

MiR-21 directly inhibits the expression of FBXO11 in melanoma, prostate cancer, and glioma, thereby promoting tumorigenesis (73). A negative correlation between miR-21 and FBXO11 expression has been observed in glioma patients, with higher FBXO11 levels associated with lower tumor grade and improved patient survival. These findings strongly suggest that FBXO11 inhibition is a key mechanism underlying the oncogenic activity of miR-21 in the progression of melanoma, glioma, and prostate cancer. Additionally, miR-621-mediated inhibition of FBXO11 enhances p53-dependent apoptosis, increasing the sensitivity of breast cancer cells to chemotherapy (74). FBXO11 typically suppresses p53 transcriptional activity by promoting p53 neddylation. Therefore, miR-621 inhibition of FBXO11 enhances p53-mediated apoptosis in breast cancer cells by blocking p53 neddylation and/or counteracting SETD8-induced p53 instability (75). These insights highlight the critical role of FBXO11 regulation in cancer progression and therapeutic response.

### 4.3 SKP2

Skp2 was identified as a target gene of miR-203, and its repression is essential for miR-203 to induce cell cycle exit and inhibit long-term cell proliferation (76). The miR-203-mediated downregulation of Skp2 enhances the stability of p21 and p27, leading to cell cycle arrest and accelerated senescence. Additionally, Skp2 was identified as a major target of the tumor suppressor miRNA miR-7, with its downregulation resulting in increased p27 levels and subsequent cell growth arrest (77). Skp2 is also a direct target of the miR-30 family in lung fibroblasts. Overexpression of Skp2 has been shown to increase lung vascular permeability and promote lung metastasis of B16 melanoma cells. Conversely, miR-30-dependent repression of Skp2 may play a critical role in suppressing lung metastasis in melanoma patients, highlighting its potential as a therapeutic target.

### 4.4 β-TrCP1

β-TrCP1 promotes the degradation of the Sp1 transcription factor, leading to reduced ADAM17 transcription. MiR-183-mediated downregulation of β-TrCP1 increases ADAM17 expression by stabilizing Sp1 (78). This mechanism suggests that miR-183 may promote tumorigenesis in U937 cells by modulating TNF-α/NF-κB signaling, whereas β-TrCP1 exhibits tumor suppressor activity. Additionally, high expression of miR-135b is associated with poor overall survival in non-small cell lung cancer (NSCLC) patients. β-TrCP1 has been identified as a direct target of miR-135b, which regulates the Hippo pathway in NSCLC cells. Downregulation of β-TrCP1 by miR-135b results in increased stability of TAZ and enhanced transcriptional activation of Hippo pathway target genes, potentially driving NSCLC progression and metastasis (75). These findings underscore the critical roles of β-TrCP1 and its regulatory miRNAs in cancer pathogenesis and progression.

### 4.5 Other MiRNA that affect F-box protein

#### 4.5.1 FBXW8

MiR-218 downregulates FBXW8 in human JEG-3 choriocarcinoma cells and promotes cell proliferation. FBXW8 mediates the ubiquitination and degradation of p27 (79) miR-218-mediated reduction of FBXW8 is expected to lead to increased p27 levels.

#### 4.5.2 FBXL20

FBXL20 was identified as a direct target of miR-3151. FBXL20 transcription is regulated by p53, which is also a target of miR-3151 (80). The multi-level downregulation of FBXL20 by miR-3151 suggests that FBXL20 may be a key target of miR-3151 in promoting carcinogenesis.

#### 4.5.3 FBXL5

FBXL5 has been identified as a potential direct target of the miR-290–295 miRNA cluster. This miRNA is enriched in mouse embryonic stem cells and promotes stem cell survival (81). The biological significance of EMT regulated by the miR-290–295 cluster in metastasis and its impact on the survival of cancer stem-like cells in human cancers deserves further investigation.

#### 4.5.4 FBXO32

FBXO32 can regulate cell apoptosis and play a tumor suppressor role. Oncogenic miR-19a/b has been shown to inhibit FBXO32 expression in cardiomyocytes (82).

## 5 lncRNAs and F-box

### 5.1 The lncRNAs regulate FBXW7 expression

LncRNA MT1JP reduced tumor size and tumor metastasis. Mechanistic analysis showed that lncRNA MT1JP recruited miR-92a-3p and upregulated FBXW7 in gastric cancer. Rescue experiments showed that downregulation of FBXW7 reversed MT1JP-induced inhibition of gastric cancer proliferation, invasion, and migration (83).

TUG1 upregulates FBXW7 expression and induces FBXW7-triggered SIRT1 ubiquitination and degradation. In addition, in cerebral ischemia-reperfusion injury, TUG1 impairs neuronal mitochondrial autophagy by targeting the TUG1/FBXW7 axis (84). Whether TUG1 regulates FBXW7 expression during carcinogenesis remains to be explored.

It has been reported that lncRNATTN-AS1 can adsorb miR-15b-5p and regulate the expression of FBXW7 in ovarian cancer. In addition, knockdown of FBXW7 can weaken the effect of TTN-AS1 upregulation on cell behavior, suggesting that TTN-AS1 exerts its biological behavior by upregulating FBXW7 in ovarian cancer cells (85).

Wang et al. reported that lncRNA CASC2 inhibited epithelial-mesenchymal transition (EMT) in liver cancer cells by targeting miR-367 and FBXW7. In addition, FBXW7 was found to be a downstream target of miR-367 in liver cancer cells. Therefore, CASC2 regulates the expression of FBXW7 by regulating miR-367 in liver cancer cells (86).

MALAT1 was identified as recruiting FBXW7 to stimulate the degradation of CRY2 and regulate the invasion and migration of trophoblast cells. In addition, FBXW7 was identified as a key downstream molecule of miR-155 in glioma cells. Notably, FBXW7 mediated the carcinogenesis of U87 and SHG139 glioma cells induced by miR-155. MALAT1 reduced cell viability by upregulating FBXW7 expression due to the downregulation of miR-155 (87).

In lung cancer cells, TINCR upregulation delayed cell invasion and proliferation by acting as a sponge for miR-544a. FBXW7 was confirmed to be a downstream target of miR-544a in lung cancer cells. In a rescue experiment, the depletion of FBXW7 eliminated the inhibition of invasion and proliferation by TINCR. That is, lncRNA TINCR exerts anti-proliferation and invasion ability in lung cancer cells by regulating the miR-544a/FBXW7 axis (88).

The expression of FER1L4 was higher in prostate cancer specimens from patients with early-stage prostate cancer. Upregulation of FER1L4 reduced proliferation and increased apoptosis of prostate cancer cells by sponging miR-92a-3p and upregulating FBXW7. Depletion of FBXW7 abolished the cell proliferation inhibition caused by FER1L4 upregulation in prostate cancer cells, indicating that FER1L4 exerts antitumor activity through the miR-92a3p/FBXW7 axis (89).

### 5.2 LncRNA SLC7A11-AS1 regulates β-TrCP1

SLC7A11-AS1 interacts with the F-box motif of β-TrCP1, preventing the ubiquitination and subsequent degradation of NRF2. By attenuating β-TrCP1-mediated NRF2 degradation, SLC7A11-AS1 reduces reactive oxygen species (ROS) levels and enhances cancer stem cell properties (90). This mechanism highlights the role of SLC7A11-AS1 in modulating NRF2 stability and its implications for cancer stem cell maintenance and oxidative stress regulation.

### 5.3 LncRNA MALAT1 targets FBXW8

FBXW8 plays a role in regulating cell growth and cell cycle progression in choriocarcinoma, with its overexpression exerting opposing effects on these processes (91). MALAT1, a long non-coding RNA, modulates its biological functions by targeting miR-218 in choriocarcinoma cells. FBXW8 has been identified as a direct target of miR-218 and is implicated in MALAT1-mediated promotion of choriocarcinoma cell proliferation (92). Thus, MALAT1 enhances cell proliferation by interacting with miR-218 and upregulating FBXW8, highlighting a critical regulatory axis in choriocarcinoma progression.

### 5.4 LncRNA PCGEM1 regulates FBXW11

LncRNA PCGEM1 plays a significant role in the occurrence and progression of various cancers by regulating multiple signaling pathways (93). PCGEM1 functions as a competing endogenous RNA (ceRNA) by sequestering miR-182 and inhibiting its expression, resulting in the upregulation of FBXW11. Additionally, PCGEM1 activates the NF-κB and β-catenin/TCF signaling pathways. Notably, the activation of these pathways by PCGEM1 can be abolished by knocking down FBXW11 (94), underscoring the critical role of FBXW11 in mediating the oncogenic effects of PCGEM1.

### 5.5 Linc01436 regulates FBXO11

miR-585 can interact with both linc01463 and FBXO11, indicating that linc01463 sequesters miR-585 and suppresses its activity, thereby indirectly upregulating FBXO11 expression in gastric cancer. In summary, linc01463 modulates the miR-585/FBXO11 axis, contributing to the progression of gastric cancer (95).

Similarly, GATA6-AS1 overexpression elevates the expression of FBXO11 and SP1 by acting as a sponge for miR-324-5p, thereby enhancing the invasion and proliferation of lung cancer cells. However, overexpression of miR-324-5p counteracts the effects of GATA6-AS1 upregulation in lung cancer. These findings suggest that lincRNA GATA6-AS1 regulates the miR-324-5p/FBXO11 axis, promoting lung cancer development (96).

### 5.6 LncRNA ODIR1 regulates FBXO25

One study demonstrated that the lncRNA RP11527N22.2, also known as osteogenic differentiation inhibitory lncRNA 1 (ODIR1), interacts with FBXO25 and promotes its degradation by recruiting Cullin3 (97). FBXO25 enhances the transcription of osterix and the expression of osteogenic markers such as osteocalcin, osteopontin, and ALP. Downregulation of ODIR1 promotes osteogenic differentiation, whereas its upregulation inhibits this process. The role of ODIR1-mediated FBXO25 degradation in tumorigenesis and cancer progression remains to be further explored (98).

### 5.7 Linc00494 regulates FBXO32

Dual luciferase reporter assays, RNA immunoprecipitation (RIP), and RNA pull-down experiments confirmed the interaction between linc00494 and NF-κB1. NF-κB1 was found to inhibit the transcription of FBXO32 by binding to its promoter region. Linc00494 upregulates NF-κB1 expression, thereby promoting the invasion, migration, and tumorigenic potential of ovarian cancer cells. In summary, linc00494 modulates the NF-κB1/FBXO32 axis and drives ovarian cancer progression (99).

## 6 Immunotherapy strategies and F-box

In recent years, several clinical trials targeting the ubiquitin-proteasome system (UPS) have promoted the progress of tumor immunotherapy. For example, a phase I/II trial (NCT06050512) evaluated the efficacy of the novel E3 ubiquitin ligase Cereblon (CRBN) modulator Mezigdomide combined with isazomib and dexamethasone in the treatment of relapsed refractory multiple myeloma (RRMM). This drug induces apoptosis of myeloma cells by enhancing ubiquitination degradation of CRBN-mediated transcription factors (such as Ikaros/Aiolos). Preliminary data show that the overall remission rate (ORR) is 55%-75%, significantly better than traditional therapies. Another phase I trial (NCT03816319) explored the application of the UAE inhibitor TAK-243 in acute myeloid leukemia (AML) and myelodysplastic syndrome (MDS). It blocks protein ubiquitination modification by inhibiting ubiquitin-activated enzyme (UAE) and interferes with cancer cell proliferation. Dose optimization and safety assessment are currently underway. In addition, Carfilzomib, as a proteasome inhibitor, was used in a phase I study of relapsed refractory solid tumors and leukemia in children (NCT02512926), and further verified the broad-spectrum potential of UPS-targeted therapy by inhibiting the degradation of ubiquitin-labeled proteins and leading to apoptosis of cancer cells. It is worth noting that the mechanism of ubiquitination is not limited to tumor treatment. Research (NCT03583177) also reveals its role in chronic kidney disease-associated muscle atrophy. It is found that the abnormal activation of E3 ligases (such as MuRF1/MAFbx) and the ubiquitin-proteasome system is closely related to muscle breakdown, which provides new ideas for the development of ubiquitination-related biomarkers and cross-disease treatment strategies. Together, these studies show that targeted ubiquitination pathways can open up innovative directions for tumor immunotherapy by directly regulating immune regulation or protein homeostasis.

### 6.1 F-box proteins as therapeutic targets in cancer

FBXW7 is the most studied tumor-suppressing F-box protein. It maintains cell cycle and genomic stability by recognizing phosphorylated substrates (such as c-Myc, CyclinE, Notch, etc.) and mediating their degradation. In a variety of tumors such as colorectal cancer and breast cancer, mutations or downregulation of the FBXW7 gene lead to abnormal accumulation of oncogene proteins, promoting tumor initiation and progression (100). For example, FBXW7 inactivation can lead to increased stability of c-Myc protein, which in turn drives abnormal cell proliferation and metabolic reprogramming.

Some F-box proteins such as Skp2 and FBXO22 have clear oncogenic functions. Skp2 promotes G1/S phase conversion by degrading p27 (cell cycle inhibitor), and its overexpression is related to the aggressiveness of lung and prostate cancer. FBXO22 is highly expressed in melanoma, which enhances VEGF-mediated angiogenesis by stabilizing HIF-1α and promotes tumor metastasis; clinical data show that FBXO22 is significantly upregulated in 74.3% of patients with malignant melanoma, and its expression level is negatively correlated with prognosis (100).

### 6.2 Drug development targeting specific F-box proteins

F-box proteins, as critical substrate-recognition components of the SCF E3 ubiquitin ligase complex, have emerged as promising therapeutic targets in cancer due to their dual roles in regulating oncoprotein degradation or stabilization. For instance, FBXW7, a well-characterized tumor suppressor, is frequently mutated or downregulated in cancers, leading to the accumulation of oncogenic substrates like c-Myc and Cyclin E, thereby driving tumor progression. Conversely, oncogenic F-box proteins such as SKP2 and FBXO22 promote cancer cell proliferation and metastasis by degrading tumor suppressors or stabilizing hypoxia-inducible factors, respectively (101). Recent advances in drug development have focused on modulating these proteins through diverse strategies. Small-molecule inhibitors targeting SKP2, such as Brusatol, disrupt SCF complex assembly to restore p27 levels, demonstrating efficacy in preclinical models of lung and prostate cancers. Additionally, proteolysis-targeting chimeras (PROTACs) have been engineered to exploit specific F-box proteins for targeted degradation. For example, the FBXO22-recruiting PROTAC 22-SLF effectively degrades FKBP12 and other oncoproteins, highlighting the potential of leveraging F-box proteins as “molecular glue” components (102). Challenges remain in achieving tissue-specific delivery and minimizing off-target effects, particularly given the ubiquitous role of SCF complexes in normal cellular homeostasis. Innovations like folate receptor-targeting chimeras (FRTACs), which selectively deliver degraders to cancer cells, may enhance precision (103). Further exploration of F-box protein interactomes and context-dependent substrate networks will be critical to advancing these therapies into clinical trials (101).

### 6.3 F-box proteins in drug resistance

1. FBXW7 holds significant potential as both a biomarker for predicting chemotherapy efficacy and a therapeutic target for overcoming chemotherapy resistance in a wide range of cancers. Its broad regulatory functions in tumorigenesis and drug resistance underscore its promising prospects for future cancer therapies.
2. Knockdown of FBXO4 results in elevated levels of the anti-apoptotic protein MCL-1, enhancing cell survival and conferring resistance to chemotherapeutic drugs in lung cancer. Conversely, overexpression of FBXO4 produces the opposite effects, sensitizing cells to chemotherapy (104). Furthermore, studies have demonstrated that combination therapies can overcome resistance to the cyclin-dependent kinase 4/6 (CDK4/6) inhibitor palbociclib in esophageal squamous cell carcinoma (ESCC) with dysregulation of the FBXO4-cyclin D1 axis (105). These findings underscore the critical role of FBXO4 in tumor progression and its involvement in resistance to anticancer therapies, highlighting its potential as a therapeutic target.
3. Depletion of FBXO5 enhances the sensitivity of human cancer cells to both chemotherapy and radiotherapy (106). Targeted inhibition of FBXO5 may represent a novel therapeutic strategy for treating cancer patients with FBXO5 overexpression. However, the oncogenic role of FBXO5 requires further investigation, particularly through the use of tissue-specific transgenic mouse models, to fully elucidate its mechanisms and potential as a therapeutic target.
4. CD44 inhibits FBXO21-mediated proteasomal degradation of P-glycoprotein (P-gp), thereby promoting P-gp-dependent multidrug resistance in a manner dependent on CD44 phosphorylation. This suggests that targeting CD44 could be an effective strategy to overcome drug resistance in cancer patients with P-gp overexpression (107). Interestingly, the survival rates of patients with high CD44 expression were significantly higher than those with low expression in the first ten years. However, after ten years, the trend reversed, with patients exhibiting high CD44 expression showing significantly lower survival rates compared to those with low expression. This dual-phase survival pattern highlights the complex and context-dependent role of CD44 in cancer progression and therapy resistance.
5. FBXO22 plays a critical role in cancer progression by regulating the degradation of key proteins. In liver cancer, FBXO22 enhances tumor formation and progression by promoting the degradation of p21, a cell cycle regulator. In malignant melanoma, FBXO22 increases tumor cell invasiveness and angiogenesis through the HIF-1α and VEGF pathways. Importantly, knockdown of FBXO22 has been shown to inhibit tumor progression and metastasis *in vivo*, highlighting its potential as a therapeutic target for melanoma treatment (100).

Additionally, FBXO22 has been implicated in modulating chemoresistance. CD147 (Basigin), a transmembrane glycoprotein of the immunoglobulin superfamily, is known to contribute to chemoresistance in various human malignancies. A study revealed that FBXO22 triggers the ubiquitination and degradation of CD147, thereby reversing cancer cells’ resistance to cisplatin (108). These findings suggest that targeting FBXO22 could be a promising strategy to overcome chemoresistance in cancer therapy.

6. EZH2, a histone lysine N-methyltransferase responsible for mediating histone methylation and transcriptional repression, plays a role in the resistance of gastric cancer cells to 5-fluorouracil (5-FU) by suppressing FBXO32 expression. FBXO32 expression is significantly downregulated in 5-FU-resistant gastric cancer cells. Interestingly, knockdown of FBXO32 enhances the cytotoxic effects of 5-FU in gastric cancer cells that have developed prior resistance to the drug (109). These findings suggest that the EZH2-FBXO32 axis is a key regulator of 5-FU resistance in gastric cancer and may represent a potential therapeutic target to overcome chemoresistance.
7. Depletion of FBXL5 and B-cell translocation gene 3 (BTG3) enhances cell invasiveness and cisplatin resistance in cervical cancer. The inhibitor of ankyrin repeats, SH3 domains, and proline-rich regions protein (iASPP), a key inducer of epithelial-mesenchymal transition (EMT), promotes cisplatin resistance by targeting FBXL5 and BTG3 through miR-20a in cervical cancer (110).

In gastric cancer, loss of FBXL5 increases cisplatin resistance by activating the Erk and p38 signaling pathways. Additionally, FBXL5 binds to RhoGDP dissociation inhibitor beta (RhoGDI2), reducing RhoGDI2-mediated cisplatin resistance in gastric cancer cells (111). These findings highlight the critical role of FBXL5 in modulating chemoresistance across different cancer types and suggest its potential as a therapeutic target to enhance chemotherapy efficacy.

8. FBXL7 regulates the expression levels of survivin through the ubiquitin-proteasome pathway. Aurora kinase A (AURKA) increases survivin protein levels by downregulating FBXL7, thereby inhibiting survivin degradation and enhancing drug resistance. This study suggests that FBXL7 contributes to drug resistance by modulating survivin expression, highlighting its potential as a therapeutic target to overcome resistance in cancer treatment (112).

In summary, F-box proteins are not only key regulators of tumorigenesis but also play a critical role in drug resistance, making them attractive targets for the development of innovative cancer therapies.

## 7 Conclusion and future perspective

As an important regulator of the ubiquitin-proteasome system, the F-box protein family plays a complex and critical role in tumor development and immune response. This review systematically integrates the dual regulatory mechanism of F-box protein with the immune regulatory network of dynamic adaptation of the immune microenvironment and summarizes the molecular mechanism of F-box protein regulating immune checkpoints. In addition, this paper systematically integrates miRNA and lncRNA to regulate F-box protein, constructs an axial regulation model of non-coding RNA-F-box protein-immune effect molecules, filling the gap in epigenetic regulation in F-box protein research. Finally, this article emphasizes the F-box protein as a target for immunotherapy sensitization. It is proposed that in the future, it is necessary to analyze the dynamic expression of F-box protein in tumor heterogeneity through single-cell sequencing, spatial transcriptome and other technologies, and develop a personalized immunotherapy plan based on F-box molecular typing. Although research on the F-box protein family has made significant progress in recent years, there are still many unknown areas to be explored. For example, the current research on F-box proteins is mainly focused on a single molecule or a single pathway. In the future, it is necessary to use multi-omics technology and high-throughput screening platforms to systematically analyze the interaction network of different F-box proteins in the tumor immune microenvironment and reveal their molecular mechanisms for regulating immune cell function, cytokine secretion and immune checkpoint expression. At the same time, we should pay attention to the expression differences and functional heterogeneity of F-box proteins in different tumor types and different immunophenotypes to provide a theoretical basis for the development of precision immunotherapy strategies. In terms of drug resistance, we need to further study the role of F-box proteins in tumor immune escape and drug resistance mechanisms, explore potential targets for overcoming immunotherapy resistance, and develop combination drug regimens for F-box proteins to reverse immunotherapy resistance and improve patient survival. In short, the F-box protein family has great research value and clinical application prospects in tumor immunoregulation. In the future, multidisciplinary cross-collaboration is needed to further explore the molecular mechanism and clinical application of F-box proteins, provide new ideas and methods for tumor immunotherapy, and ultimately benefit the majority of cancer patients.
